# Nanoceramic Composites for Nuclear Radiation Attenuation

**DOI:** 10.3390/ma15010262

**Published:** 2021-12-30

**Authors:** Shankar A. Hallad, Nagaraj R. Banapurmath, Avinash S. Bhadrakali, Arun Y. Patil, Anand M. Hunashyal, Sharanabasava V. Ganachari, T. M. Yunus Khan, Irfan Anjum Badruddin, Manzoore Elahi M. Soudagar, Sarfaraz Kamangar

**Affiliations:** 1 Centre of Excellence in Material Science, School of Mechanical Engineering, KLE Technological University, B. V. Bhoomaraddi Campus Vidyanagar, Hubballi 580031, Karnataka, India; bhadrakaliavinash@gmail.com (A.S.B.); patilarun7@gmail.com (A.Y.P.); amhunashyal@kletech.ac.in (A.M.H.); 2 School of Civil Engineering, KLE Technological University, B. V. Bhoomaraddi Campus Vidyanagar, Hubballi 580031, Karnataka, India; 3 School of Advanced Sciences, KLE Technological University, Vidyanagar, Hubballi 580031, Karnataka, India; sharanu14@gmail.com; 4 Department of Mechanical Engineering, College of Engineering, King Khalid University, Abha 61421, Saudi Arabia; yunus.tatagar@gmail.com (T.M.Y.K.); magami.irfan@gmail.com (I.A.B.); sarfaraz.kamangar@gmail.com (S.K.); 5 Department of Mechanical Engineering, School of Technology, Glocal University, Delhi-Yamunotri Marg, SH-57, Mirzapur Pole, Saharanpur 247121, Uttar Pradesh, India

**Keywords:** lead oxide, radiation shielding, cement composites, gamma attenuation, flexure strength, deflection

## Abstract

The development of radiation attenuation materials with lean cross-sections is the need of the hour. However, the inherent threat of radiations accompanying these processes is of major concern. Thus, in an attempt to shield unnecessary radiations, several novel materials have been fabricated alongside the conventional materials available. Yet, there is a need for cost-effective, efficient shielding materials that have good mechanical strength and effective shielding properties. The present work investigates ceramic composite behaviors and radiation shielding capacity reinforced with lead oxide nano-powder. Developed nano-lead-based cement composites were subjected to mechanical tests to determine flexural and compressive strengths to check their suitability for structural applications. Further, the gamma attenuation test of the composites was conducted to determine their neutron absorption capacity. The addition of nano-leadoxide in the control beams was varied from 0.7 to 0.95 and 1 wt.% of the ceramic matrix. The percentage of nano-leadoxide that gives the best results in both enhanced properties and economic aspects was determined to be 0.6 wt.% of the cement.

## 1. Introduction

Lead can effectively attenuate certain types of radiation, such as X-rays, γ radiation, and neutron radiation. This is mainly due to its high density and high atomic number. The high density (9.53 g/cm^3^) of lead is due to the combination of its relatively small atomic size and high atomic mass, atomic number (82) and molecular weight (223.2 g/mol). This results in relatively more electrons and a smaller bond length. Thus, due to a greater number of electrons, lead can effectively block high-energy electromagnetic radiations such as X-rays and γ-radiation by absorption and scattering of the photons.

The applications of lead as a radiation shielding material, current safety issues related to lead, and recent developments of new lead-free shielding materials in nuclear medicine have been reported in the literature [1]. Different radiation shielding materials have been produced to safeguard humans and their surroundings from the destructive impact of radiation [2]. Materials used for gamma radiation should have high density, such as concrete or lead [3]. Heavy materials are known to have high abilities in the attenuation of gamma rays, which is the most important characteristic of a radiation shielding material for radiation protection [4].

However, lead is not particularly effective in absorbing radiations consisting of neutrons. Thus, incorporating a neutron shielding material is essential in developing the shielding material [5]. Unlike other types of ionizing radiations, shielding neutrons is a relatively complicated process and requires materials with heavy atomic nuclei—neutron shielding results in secondary β and γ radiations due to the mechanism of the shielding process. However, concrete, a heavyweight material, is used to shield neutron radiations in both medical and structural applications. The use of heavy natural aggregates, such as barite and magnetite, increases the density of normal concrete and improves its radiation shielding properties. However, the increased density sets several limitations in its utilization for structural applications. Conventional Portland cement was used in developing the new composite material [6].

The practical application of composite materials in radiation shielding was realized long ago. However, due to limitations such as cost, the newly developed materials have not been implemented. 

Lead oxide nano-powder or nanoparticles are nanostructured magnetic particles with high spherical or faceted surface areas. These are typically 20 to 30 nm in size and have a specific surface area (SSA) greater than 5 m^2^/g. Nambiar et al. reported a lead-based polymer composite for radiation shielding applications using a balling milling process to prepare the nano-lead powder [7]. Polymer composite materials were developed using graded shield material that contains heavy atoms impregnated within hydrogen-rich polymer matrix along with other micro or nanomaterial such as boron, metal oxides, graphitic fibers, and metal whiskers. Kim et al. developed nano-W dispersed gamma radiation shielding materials [8]. A polymer nano-composite-based novel multifunctional neutron shielding material was designed and fabricated by Gözde İrim et al. [9]. Mortazavi et al. fabricated high-density borated polyethylene nanocomposites as a neutron shield [10]. Further, the enhancement of nuclear radiation shielding properties of nano-B4C, nano-BN dispersed polymer nanocomposites, were investigated by Kim et al. [11]. Saidova, Z. et al., reported on cement-based composites with a complex additive of chrysotile nanofibers and carbon black. Addition of optimized percentage of chrysotile and carbon black in cement results in increase of 31.9% compression strength and a 26.7% flexural strength of cement composite [12].

However, it may be noted that limited work has been reported on the development of ceramic-based matrices incorporated with nanoparticles for nuclear radiation shielding applications. Hence, the objective of the present work is to develop and characterize novel lead oxide nano-powder-based ceramic composite materials for nuclear radiation shielding structural applications.

## 2. Experimental Approach

This section discusses the materials used and the procedure implemented in developing the nanocomposites and testing the nanocomposites as per ASTM standards.

### 2.1. Materials and Methods

Properties of lead oxide nano-powder used in the study are presented in Table 1. Lead oxide used in the development of the specimen was industrial-grade nano-powder with purity levels greater than 99.9%. Uniform dispersion of the nanoparticles against agglomeration is essential and regarded as the first step in preparing nanocomposites. To achieve the same, the nanoparticles were probe sonicated for 20 min with water as the dispersion media. In the meantime, the appropriate cement-to-water ratio was weighed (less water was used for dispersion during sonication).

### 2.2. Synthesis of Lead Oxide (PbO) Nanoparticles

To create PbO nanoparticles, a chemical synthesis method was employed. The micro-level lead oxide was heated to 90 °C with de-ionized water to make the 60 mL solution of 1.0 M lead acetate trihydrate. To dissolve this solution, it was added to a 50 mL beaker containing 19 M NaOH and vigorously stirred. The color of the solution briefly changed from hazy to peach to bright orange-red after the addition of lead acetate. Once the stirring stopped, the precipitate was allowed to settle for a short time. After the supernatant had been decanted using a funnel and cleaned with distilled water, it was dehydrated in an overnight drying oven set at 80 °C for several hours. The sample was gently crushed in a pestle and mortar to ensure that it was removed from the final product. The characterization of the material was carried out to validate the presence of lead oxide nanoparticles.

### 2.3. Preparation of Specimens

**Figure 1:**
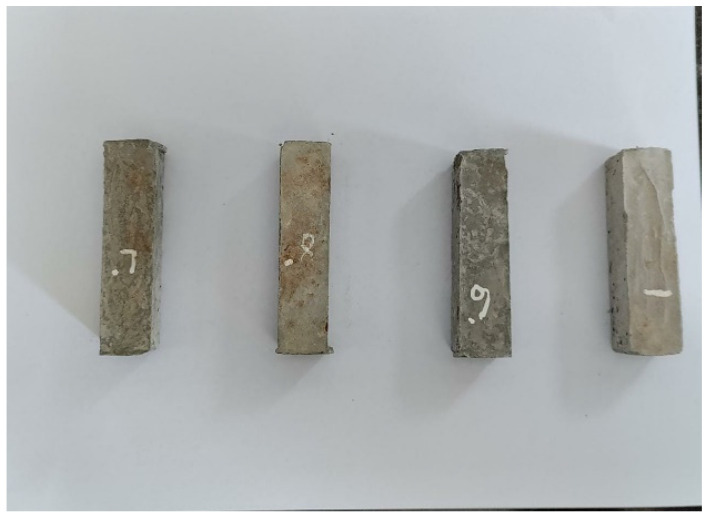
Samples developed.

**Figure 2:**
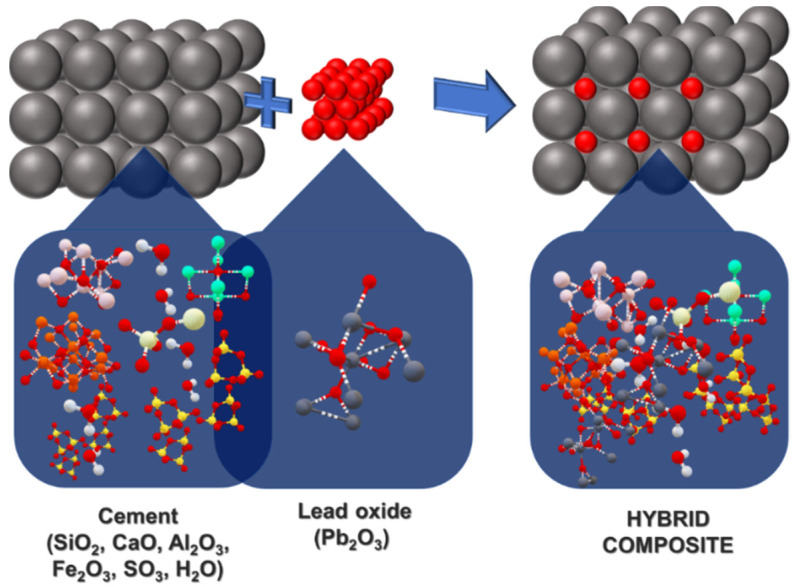
Pictorial representation of the development of hybrid nanocomposites.

At concentrations of 0.7, 0.8, 0.9, 0.95, and 1 wt.%, nano-Pb_2_O_3_ (lead oxide) was used as a filler material in the cement matrix, and it was shown to be effective. Water was used to make approximately one-third of the total weight of the cement matrix. It was necessary to utilize a steel mold to cast the specimens, which were 20 mm × 20 mm × 80 mm in size. Specimens were cured for 28 days before being un-molded and tested for characterization and performance. Figure 1 shows the actual samples developed in the lab using the varied percentage of nano-lead reinforced in the cement matrix. Figure 2 depicts the development of the hybrid nanocomposite.

To achieve the desired results, various specified amounts of dispersed lead oxide nano-powder were added to the water-mixed ceramic matrix, as indicated in Table 2.

### 2.4. Experimental Set-Up

**Figure 3:**
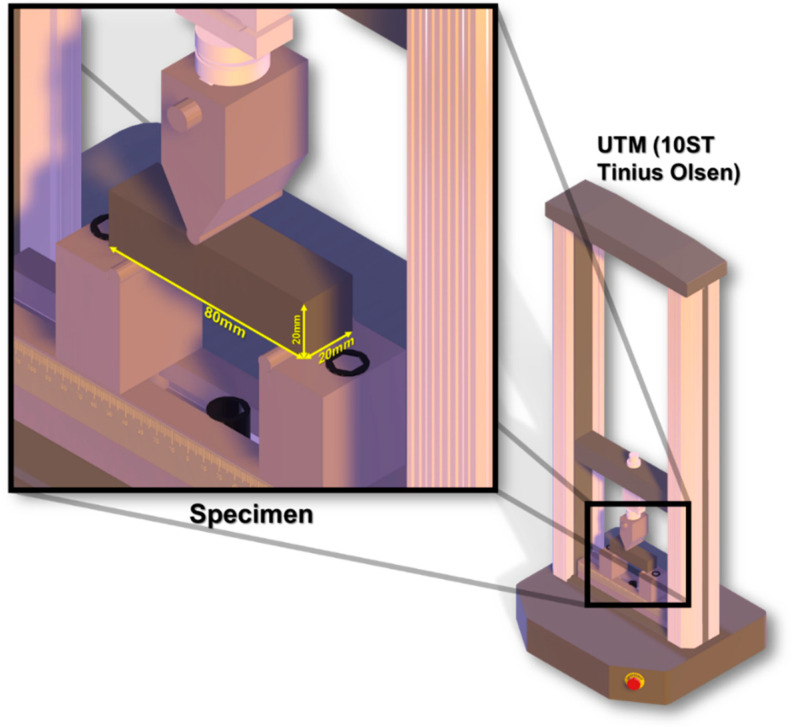
Equipment used for three-point load testing.

**Figure 4:**
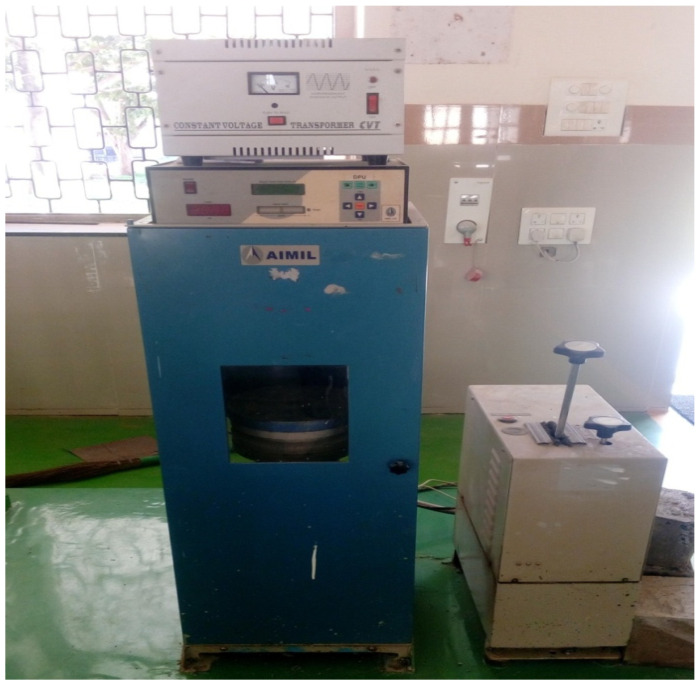
Equipment used for compression test.

The mechanical properties of the developed specimens were evaluated by flexure test and compression load test. Specimen of size 20 mm × 20 mm × 80 mm were tested using three-point loading. A hydraulic closed-loop testing machine, Aimil Ltd. (New Delhi, India) was used. The equipment used for the three-point load test is shown in Figure 3, with the sample placement for the three-point load set-up. Figure 4 shows the equipment Aimil Ltd., New Delhi used for the compression test.

## 3. Results and Discussions

This section presents the compressive results obtained to study the behavior of nano-cement composite specimens developed.

### 3.1. Three-Point Bending Test

**Figure 5:**
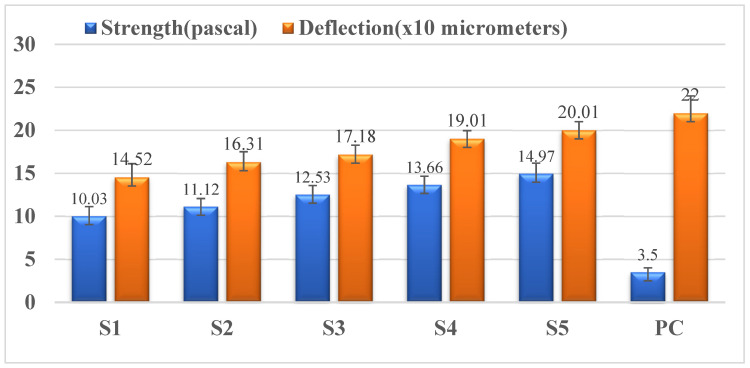
Variation of strength and deflection for different samples.

This test enables us to determine the best percentage of lead oxide to be used as reinforcement in the cement matrix based on the flexure strength of the developed material. Hence, the flexural behavior of nano-lead oxide-reinforced cement composites was investigated. The newly developed beams were subjected to three-point loading to determine their strength-deflection behavioral properties. The outcome of the tests conducted was plotted against the flexural properties of plain cement beams to understand the improvements for structural applications. The load v/s deflection of the specimen is shown in Figure 5. From the figure, it follows that the flexure strength of the newly developed material increases as the percentage of Pb_2_O_3_ in the ceramic matrix increases. The maximum flexure strength while considering the economic aspects of the developed specimen was found to be 14.97 MPa. It is also evident that the brittleness of the developed specimens decreased as the percentage of Pb_2_O_3_ increased in the cement matrix. The increase in the strength may be due to the incorporation of DLC (diamond-like carbon) material into the cement matrix that belongs to the family of carbon elements [13]. The microstructure design of the composite composed of materials with different elastic moduli is also a factor for the increase in the strength of the modified composites [14].

### 3.2. Compression Test

**Figure 6:**
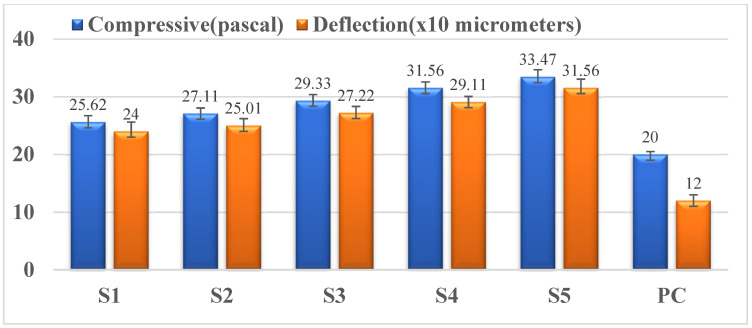
Variation of strength v/s deflection in compression.

This test enables the evaluation of the compressive strength of the developed novel material. This helps in deciding the optimal percentage of lead oxide nano-powder reinforcement in the ceramic matrix that enhances the compressive strength compared to plain cement for structural applications. It is evident from Figure 6 that the compressive strength of the novel material increases as the percentage of lead oxide nano-powder in the cement matrix increases. The maximum compressive strength while considering economic aspects was found to be 33.47 Pa. The compressive strength was directly proportional to the constituents that form pore structure in the cementitious matrix [15]. From the SEM image depicted in Figure 6, an increase in the porosity can be observed between the interfacial nanoparticles. The increase in the porosity has influenced the mechanical property under study.

### 3.3. Gamma Attenuation

Lead is very effective in shielding nuclear radiation. However, its individual effects regarding absorption of radiation for gamma radiations when used as reinforcement in the cement-based matrix was investigated. The following are the test results obtained for gamma attenuation. From Table 3, it is found that the S5 specimen had a better neutron absorption capacity than plain cement. The mass of plain cement was 53.51 gm, and the mass of the S5 specimen was 55.92 gm.

The S5 had higher mass when compared to plain cement; its density increased, which in turn increased its specific gravity and absorption capacity. Hence, S5 showed higher neutron absorption capacity.

**Figure 7:**
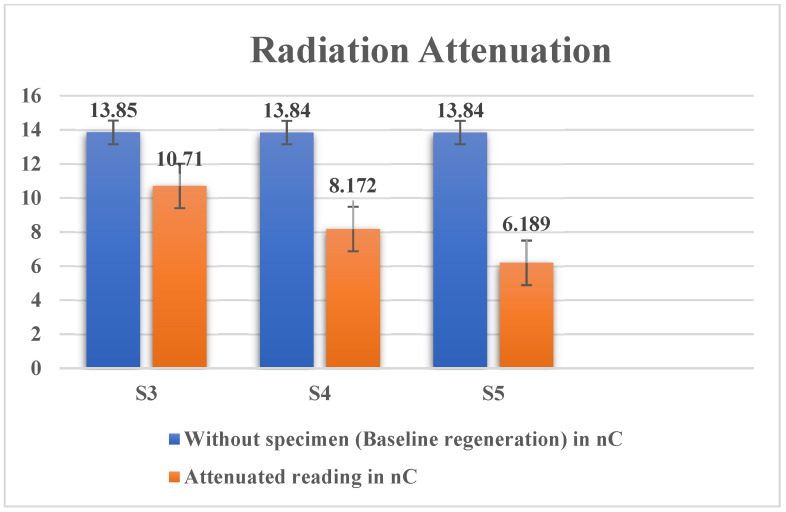
Attenuation for specimens with and without lead oxide reinforcement.

Figure 7 shows the attenuation variation for the specimens with and without lead oxide reinforcement. As the nano-lead oxide filler dosage increased in the cement matrix, the attenuation decreased. Compared to the plane specimen with no filler, S5 showed a 55.34% attenuation rate. The increase in the radiation attenuation was due to the increase in density of the nanocomposite from the nano-lead oxide [16].

### 3.4. SEM and EDAX Analysis 

**Figure 8:**
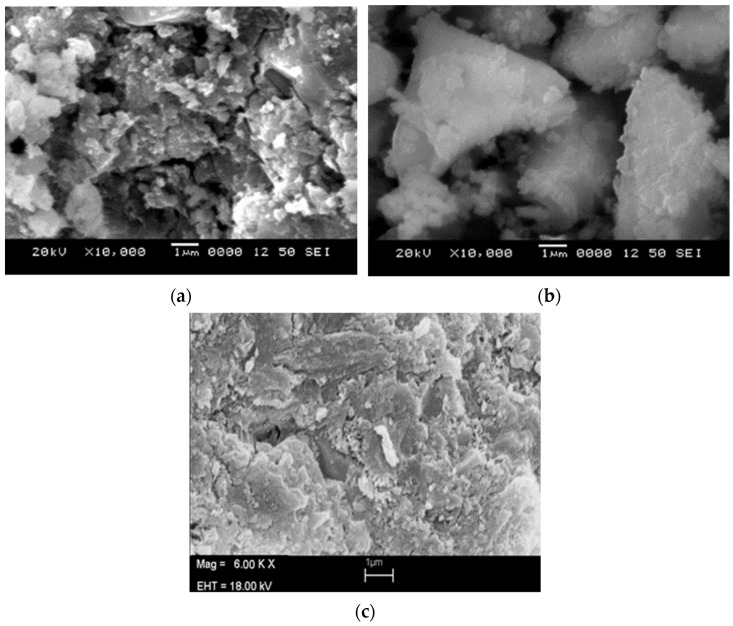
(**a**) 0.4% Pb_2_O_3_ (S4), (**b**) 0.6% Pb_2_O_3_ (S5), (**c**) Plain cement (PC).

Figure 8a,b shows SEM images of modified cement reinforced with lead oxide nano-powder; a uniform distribution of reinforcement is evident. Figure 8c is an SEM image of plain cement. From EDAX analysis, it can be inferred that there is a better distribution of lead oxide nano-powder in S5. The EDAX results are summarized in Table 4 and Table 5 below for S4 and S5.

## 4. Simulation and Modeling

In the last two decades, a module known as simulation has gained lot of importance for evidence in the Industry Internet of Things (IIOT) [17,18,19,20]. The simulation covers software tools with a background such as the Finite Element method, molecular dynamics, solid mechanics. Ansys, J-OCTA, and Material studio are the current software tools in use for the analysis of newer materials developed [21]. In this work, the ANSYS workbench has been considered as a tool to validate the experimental results.

### 4.1. Simulation Method

**Figure 9:**
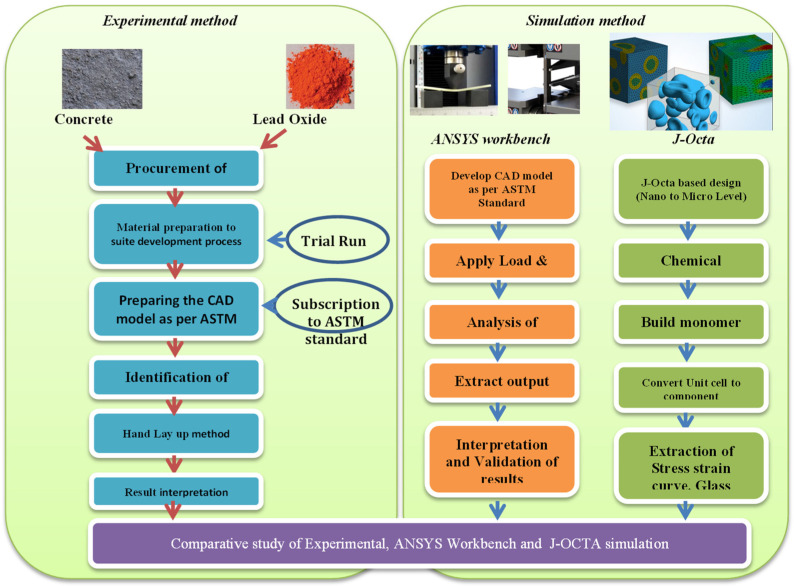
Process map for simulation in experimental work.

Among simulation software, the ANSYS workbench is currently the leading tool in industry, able to solve multidisciplinary-related problems. The roadmap for solving the current problem was considered with the following process map, as shown in Figure 9.

### 4.2. Simulation Process

The current simulation tool deals with geometry models for flexural strength tests, leading to the estimation of deformation [22]. For better correlation purposes, one case with 1% lead oxide reinforcement in cement matrix condition was considered with several iterations to converge the solution along with validation. It is possible to model the air-pockets in the simulation tool using Ansys. However, in this work, the effect of air pockets is not considered as there will already be coarse and fine aggregates that would have created gaps and, subsequently, air pockets. In this way, air pockets are retained in the mix.

#### 4.2.1. Material Properties

Concrete and lead oxide material properties are shown in Table 6.

#### 4.2.2. Geometry

**Figure 10:**
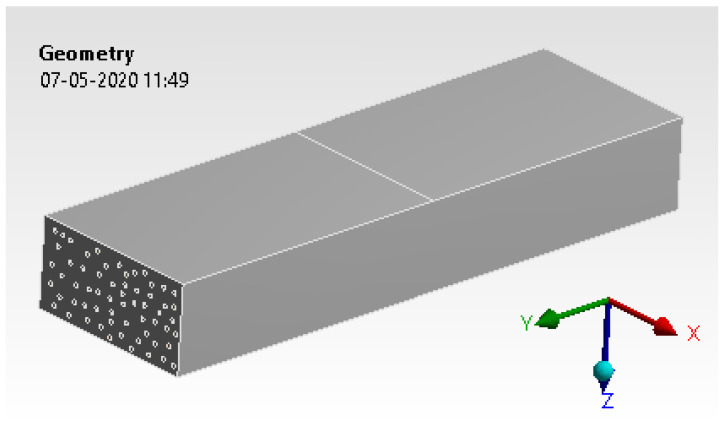
Concrete and lead oxide.

The CAD model was developed with the relevant size and shape, as mentioned in the earlier section. The model is shown in Figure 10.

#### 4.2.3. Contact Generation

**Figure 11:**
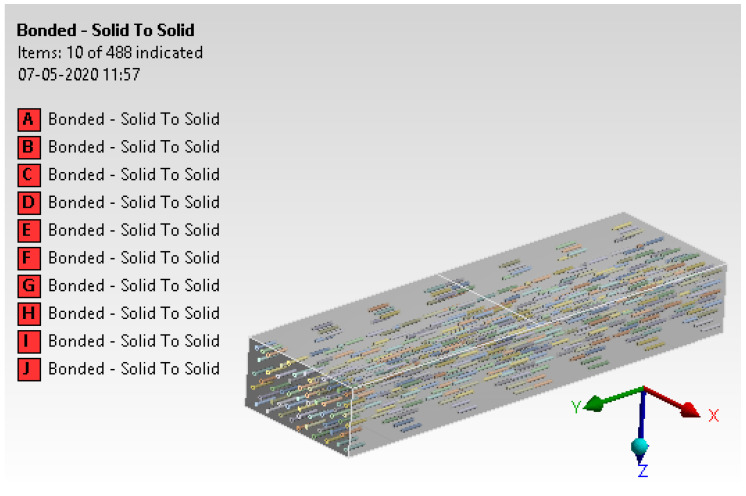
Contact generation.

Contact generation between lead oxide and concrete is assigned with ‘Bonded’ contact. Each of these contacts was considered with the ‘Pure penalty’ approach [23,24]. The details of contact generation are illustrated in Figure 11.

#### 4.2.4. Mesh Generation

**Figure 12:**
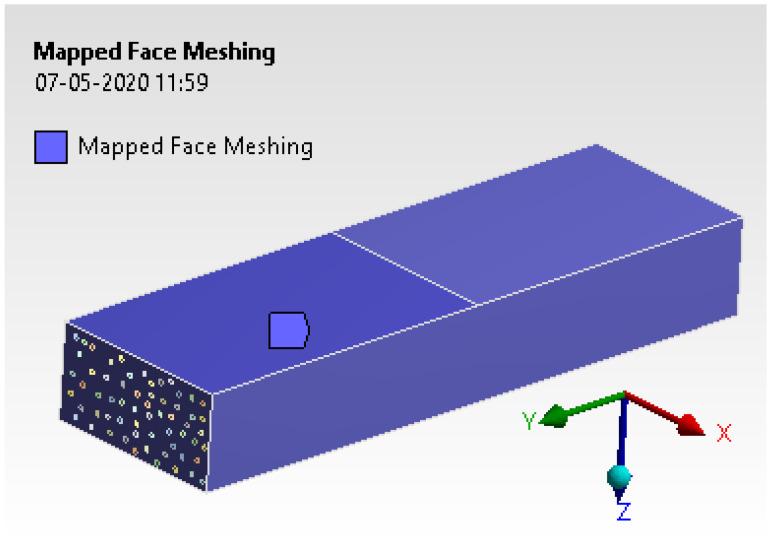
Mapped face meshing.

Mesh generation was assigned with mapped face meshing to arrive at the near-exact solution. The h-type and p-type methods were used to analyze the results. The process uses a tetrahedron element with 10 nodes of the second-order condition [25]. Figure 12 provides fine mesh conditions with checking other converging conditions. The entire model was solved for 326,070 elements and 856,686 nodes.

#### 4.2.5. Loads and Boundary Conditions

**Figure 13:**
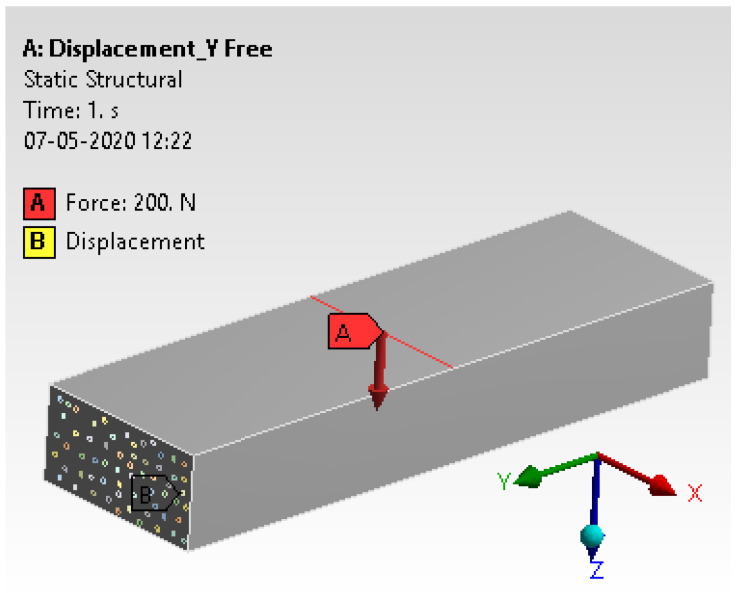
Loading details and boundary conditions.

The details were fetched from experimental analysis to arrive at loads and boundary conditions. The three-point bend test was considered with free displacement in the y-direction while the other two directions were fixed. Figure 13 illustrates the loading details and boundary conditions.

#### 4.2.6. Results and Interpretation

**Figure 14:**
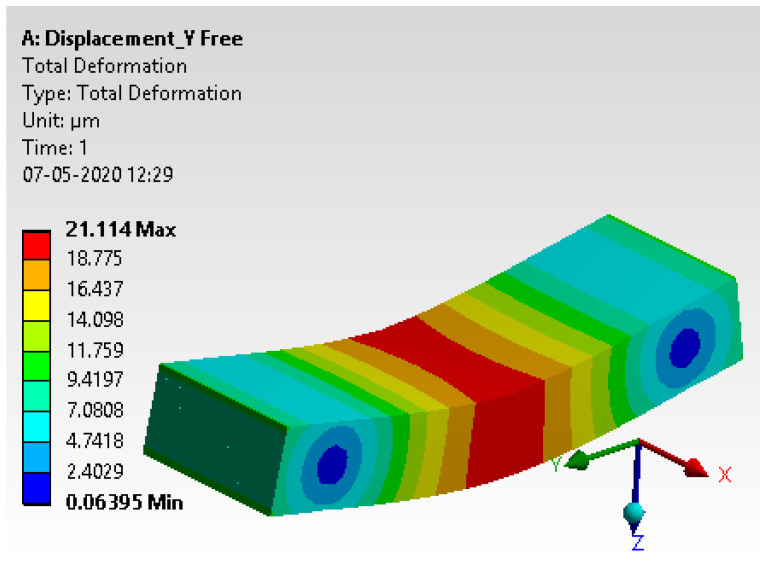
Total deformation.

The total deformation has been extracted from the analysis, and details are discussed in Figure 14.

#### 4.2.7. Comparative Study with Validation

Experimental method results were then compared with simulation results with the help of tabular data, as shown in Table 7. The comparative study reveals a percentage error of 5.21, which is well accepted within industry-standard as for composite materials, the acceptable error range is 20%.

## 5. Conclusions

Lead is very effective in shielding nuclear radiation. However, its individual effects regarding absorption of gamma radiation when used as reinforcement along with other elements were investigated. 

An attempt was made using materials such as lead fibers, steel fibers, and the combination of both as reinforcements for the cementitious matrix to shield nuclear radiation. The results were promising, as the newly developed material exhibited enhanced mechanical and shielding properties. The lead-zinc granulated slag was used as an alternative for sand in cement matrix to block radiation with the thinnest section of concrete, as compared to the conventional concrete sections. The results showed that the produced concrete demonstrates better radiation attenuation properties with thinner thickness compared to conventional concrete. Thus, it can be intuitively expected that the new material exhibits enhanced radiation absorption properties. 

From the conducted tests, it can be summarized that the properties of the newly developed radiation shielding material are better in terms of radiation shielding ability, flexural strength, and compression strength. Sample S5 showed a higher compressive strength of 33.47 pascals and deflection of 31.56 compared to PC. Compressive strength increased by 67.35%, and deformation increased by 163%, respectively. The S5 sample showed a higher radiation attenuation of 123.72%. Thus, the newly developed material could be suitable for structural applications replacing concrete. A higher dosage of lead oxide nano-powder reinforcement into the cement matrix with improved dispersion technique for improved shielding properties of the developed composite requires continued and sustained research.

## Figures and Tables

**Table 1:** Properties of the lead oxide nano-powder used in the study.

Parameters	Properties
Melting point	888 °C
Purity	99.9%
Molecular weight	223.2 g/mol
Density	9.53 g/cm^3^
Atomic number	82
Appearance	Red or yellow crystalline
Morphology	Solid spherical
Particle size	20–30 nm

**Table 2:** Details of the test specimen for mechanical test.

Sample No	Specimen Reference	Constituents	Dimensions	% of Pb_2_O_3_
1	S1	Plain cement+ Pb_2_O_3_	20 mm × 20 mm × 80 mm	0.7
2	S2			0.8
3	S3			0.9
4	S4			0.95
5	S5			1
6	PC			Nil

**Table 3:** Estimated gamma attenuation levels.

Sl. No.	Baseline Data Generation (without Specimen)	Attenuated Reading in mm	Reading in mm
1	13.85	10.71 (S3)	2.2671
2	13.84	8.172 (S4)	4.0953
3	13.84	6.189 (S5)	5.5281

**Table 4:** EDAX results, 0.4% Pb_2_O_3_ (S4).

Element	Weight (%)	Atomic (%)	Error (%)
C K	2.67	5.60	18.75
O K	32.35	50.98	11.53
Mg K	0.78	0.81	16.43
Al K	3.06	2.86	8.69
Si K	10.17	9.13	5.32
S K	0.81	0.63	13.80
Pb M	1.77	0.22	13.86
Ca K	44.49	27.99	2.23
Fe K	3.90	1.76	9.27

**Table 5:** EDAX results,0.6% Pb_2_O_3_(S5).

Element	Weight (%)	Atomic (%)	Error (%)
C K	3.23	6.25	14.81
O K	40.77	59.27	10.76
Mg K	0.67	0.64	15.51
Al K	3.62	3.12	7.25
Si K	7.26	6.01	5.31
S K	0.58	0.42	13.77
Pb M	1.50	0.17	12.43
Ca K	39.51	22.93	1.98
Fe K	2.86	1.19	9.05

**Table 6:** Material properties for concrete and lead oxide.

Sl. No	Material	Young’s Modulus	Poisson’s Ratio (MPa)	Density (kg/m^3^)
1	Concrete	30 × 103	0.18	2300
2	Lead Oxide	16 × 103	0.38	9530

**Table 7:** Comparative study.

Description	Experimental Method	Simulation Method	% of Error
Total deformation	20.01	21.11	5.21

## Data Availability

The data presented in this study are available upon request from the corresponding author.
